# Developments in laboratory automation–just a matter of time

**DOI:** 10.1155/S1463924694000192

**Published:** 1994

**Authors:** Peter B. Stockwell

**Affiliations:** Visiling Professor in Deparlment of Environmental Sciences University of Plymoulh; and Analytical Ltd 84 Chaucer Business Park Watery Lane, Kemsing, Sevenoaks, Kent 7N15 6Q7 UK


Journal of Automatic Chemistry, Vol. 16, No. 5 (September–October 1994), pp. 155–160

### Developments in laboratory automation—just a matter of time*

**Peter B. Stockwell**

*Visiting Professor in Department of Environmental Sciences, University of Plymouth; and Analytical Ltd, 84 Chaucer Business Park, Watery Lane, Kemsing, Sevenoaks, Kent 7N15 6QJ, UK*

Automatic analysis is well established as a subject area, but there is still no adequate definition of the area that the subject embraces. The International Union of Pure and Applied Chemistry (IUPAC), has sought, through its Commission of Analytical Nomenclature, to offer rigorous definitions and to provide a common international terminology. Automation is defined as: 'the use of combinations of mechanical and instrumental devices to replace, refine, extend or supplement human effort and facilities in the performance of a given process, in which at least one major operation is controlled without human intervention, by a feedback mechanism'. Mechanization, on the other hand, is defined as: 'the use of mechanical devices to replace, refine, extend or supplement human effort'. The distinction between the two terms is quite clear according to IUPAC, insofar as automation describes systems that involve a feedback loop. Whilst this is quite logical, it is not particularly useful because a feedback unit is not vital to an analytical chemist. The major thrust for research and development in automation and/or mechanization is largely twofold:

(1) To improve the cost-effectiveness in discharging large analytical workloads, especially those of a repetitive nature.

(2) To improve the method performance, notably precision, in these circumstances. The advances made have been commonly called 'automation'.

Microprocessors, computers and robots are in themselves only subsets of the general field of automation, whilst sample preparation is the major bottleneck in analysis. Little attention has been paid to this particular area.

The principal operations involved in a chemical analysis are set out in figure 1.

In considering the benefits of automating an analysis, each step in figure 1 must be taken into account. Sampling requirements will often not be amenable to automation and the task of reporting the result to the end-user may not be clearly defined. Analysts are frequently not involved in the choice of sample nor in the reporting of the result and it is important that they should be. For example, a sample which is not representative of the material it comes from will be of no real value, just as the reporting of a result without full justification of its precision is pointless. Very few publications on automatic analysis have embraced all the steps outlined in figure 1.

The reasons for installing automatic analysers to replace manual methods are many and varied, and their priority will be related to the nature of the organization of which the laboratory is part. The principal motivation for most organizations is economics; the effect of automating can be assessed, both as an internal laboratory study, or for the company as a whole, particularly in the industrial environment. When the economic case for automation is made, the true cost of manual and automatic analysis must be calculated to determine whether the additional output, or staff savings, from automatic analysis outweighs the cost of the automatic equipment over a reasonable period.

To the first approximation, the costs of a manual analysis can be identified as shown in equation 1.

Equation 1. Costs of analysis—manual procedure,

$$\text{Cost} = F_m(A_1T_1 + A_2T_2 + A_3T_3 + A_4T_4 + A_5T_5 \ldots)$$

$F_m$ = Manual tariff factor converts to a monetary unit
$A_i$ = Activity, i.e. staff involvement
$A_i$ = 1 when staff completely involved
$A_i$ = Reduced significantly when staff are simply loading a sample onto a tray or drying a sample in an oven overnight
$T_i$ = Time involved in activity

Total cost reduced either by reducing Time or Activity.

The $A$ factors for each stage represent the operator's time—for drying and incubation it is very small, but for weighing, or any other process involving a substantial amount of the analyst's time, it is high. $F$ is a tariff factor, incorporating staff and overhead costs, to convert the

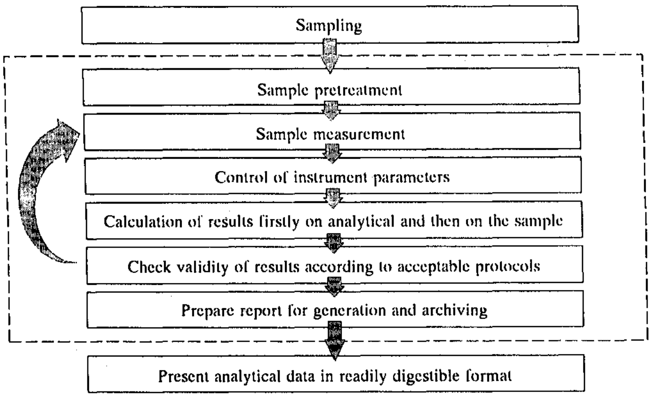

*Figure 1. The steps involved in analysis. The steps enclosed within the dashed lines are normally under control of the analyst. Those outside of the dashed lines are not.*

* *Inaugural lecture: University of Plymouth.*

0142-0453/94 $10.00 © 1994 Taylor & Francis Ltd.

activity and time factors into the real cost in cash terms. From equation 1 it can readily be shown that there are three ways of reducing the cost of an analysis. Firstly, reducing the analysis time—this is often put forward as a prime justification for automation. The reduction in time also leads to an increase in analytical capacity, and, in some cases, this capacity can be of value in providing additional revenue. Secondly, the costs can also be reduced by lowering the $A$ factor, even at the expense of increasing the time required for the task. For the same analysis carried out fully automatically the cost is given by equation 2.

Equation 2. Cost of analysis—fully automatic procedure.

$$\text{Cost} = F_A(A_R T_R + A_S T_S)$$

$F_A$ = Automatic tariff factor converts to a monetary value
$A$ = Activity of staff involvement
$T$ = Time involved in activity
R = Running automatic system
S = Service needs for preventative and ongoing maintenance

If the method is not fully automatic, any stage which remains manual can be costed in equation 1 and carried forward into equation 2. It is important to include the value of the maintenance required to keep the automatic system running smoothly and reliably. Of course, the design of an automatic instrument should take this factor into account.

Time is therefore a fundamental part of laboratory automation and in the area of computer data processing the rapid changes that have taken place over the last 25 years are quite startling. Results from analytical measurements can now almost be instantaneous. There is a ready access to computer power, commercial interface cards and even bespoke software.

Figure 2 provides a schematic representation of the processes involved in the author's work circa 1960s. The software had to be written and checked for errors prior to any data being analysed. Often the user was frustrated for days by simple syntax errors or even a tear in the paper tape. It could be a very lengthy process even to get a properly operating programme not understanding correct data collection or calculation.

In the 1970s progress was made along several fronts, for example models were available to transfer information over the telephone lines and hence cut out postal delays. In addition, instrumental data could be provided both as magnetic or paper tape output. Delays also occurred in this regime, but significant time improvements resulted.

In the 1980s the advent of microprocessors speeded up the processes still further, but efforts had to be made to provide programs to collect and compute data. Today it is even possible to get a data handling system up and running in a few days and to ensure results are computed on-line. In the area of computing, then, the time element has been greatly reduced.

### Education

Automation, however, is a great deal more than computer power and control. It involves many disciplines and the education of students requires co-operation with industry and access to past experiences. In this particular area the co-operation between my own company and the University of Plymouth, especially the Department of Environmental Sciences, is particularly valuable. Such exchanges are a vital part of the UK's recovery and more effort must be made to expand on such schemes as the 'Case Award' and 'Link' programmes.

The analysis of trace elements, in particular mercury, provides a real world example of laboratory automation. It involves consideration of all the stages of the analytical procedure and provides a unique concept. Automation where it is required, and simple mechanization when this is more economical and meets the needs of the user.

Measurement techniques used are the subject of a continuous research and development activity within the Environmental Chemistry Department. This research has integrated contributions from many other groups—for example, Queen's University Belfast (development of optical filters), the University of Gent (adsorption/desorption techniques for mercury) and also Yorkshire Water LabServices.

The analysis of mercury presents the analyst with a difficult problem, the levels in nature are very low and the responses from the standard trace elemental techniques to direct measurement are insufficient for the task in hand. Normal nebulization techniques for atomic absorption, inductively coupled plasma and direct current plasma spectroscopy are extremely inefficient, normally only 1–2%. The use of a vapour generation technique, however, has been the subject of considerable research and thus effectively improves the overall efficiency of the measurement approach by at least 50 times.

The addition of a specific atomic fluorescence spectrometer, effectively designed to provide a single element analyser, further enhances the levels of performance available.

### *Analysis of mercury in rivers and effluents*

A specific fluorescence detection system has been designed and developed to provide fully supported analytical systems for routine analysis of total mercury at low levels. The fluorescence approach provides a wide linear dynamic range and extremely low detection limits. P S Analytical's Merlin Plus System provides a fully automated system which will analyse results at a rate of 40 plus per hour. This is due to the attention to detail in the design of the PSA Merlin and its automation, coupled to the inherent features of the fluorescence technique.

The Merlin Plus System, shown in figure 3, has been specifically tailored for the needs of the market place and the equipment used in its concept evolved from previous designs and feedback from existing users. The preferred configuration encompasses a random access autosampler linked to a vapour generator system. The mercury vapour produced by the reaction with acidified tin (II) chloride is transferred into the PSA Merlin fluorescence detector

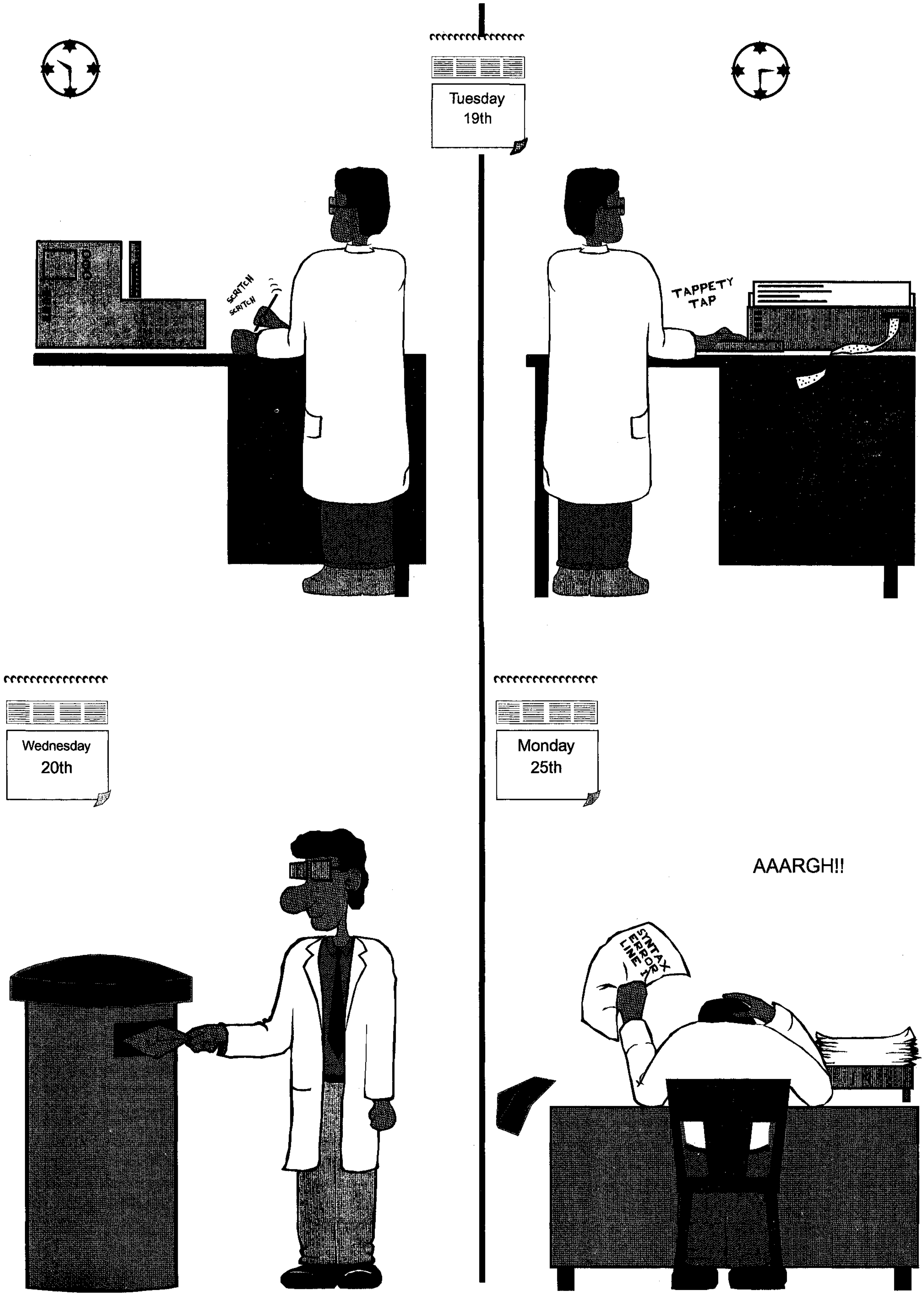

*Figure 2. Computer operations circa 1965–1969. Illustrating manual data collection, conversion to paper tape media, transmission to computer bureau and the, all too often occurring, end results.*

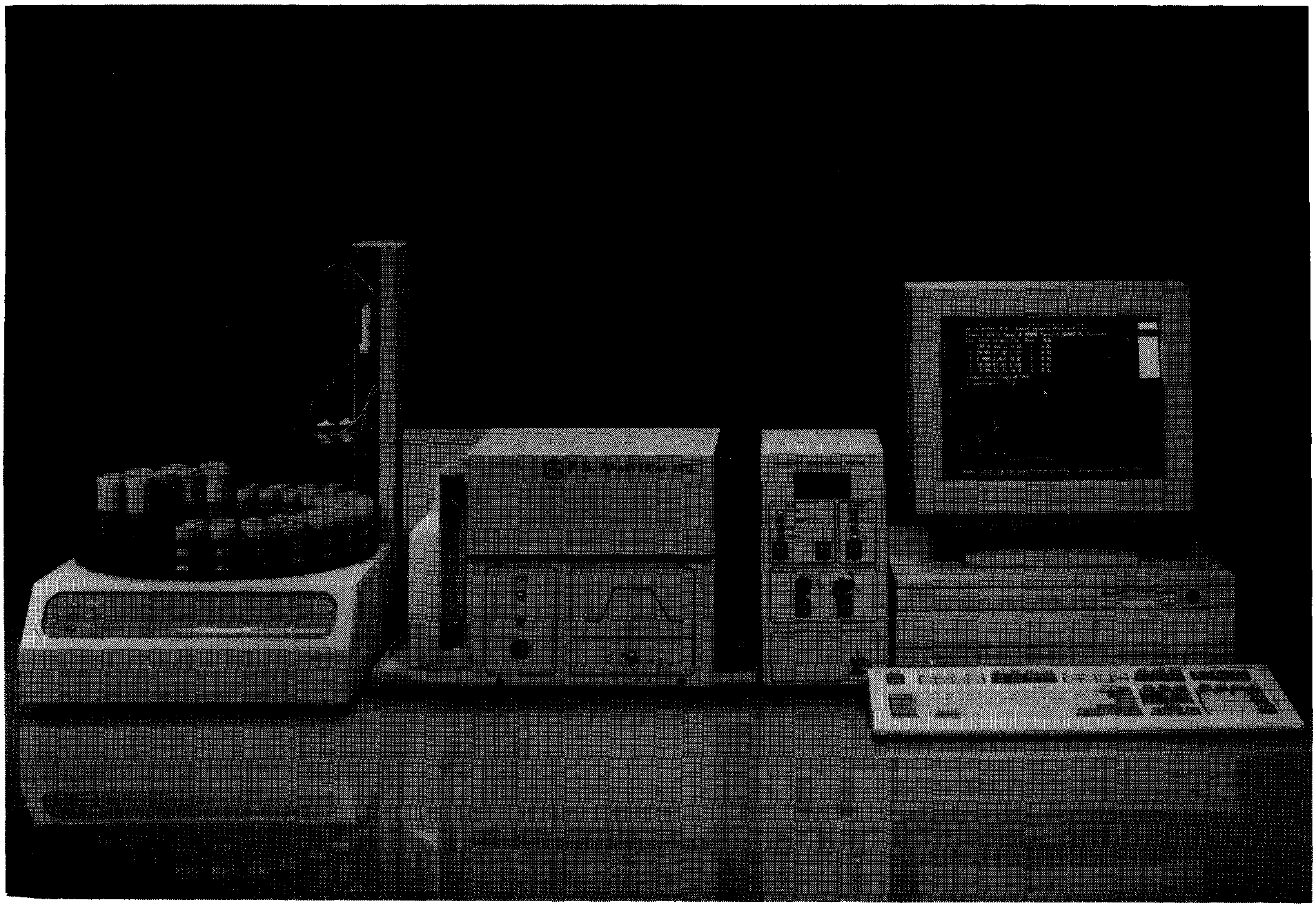

*Figure 3. Merlin Plus System for low level mercury measurements. From left to right: autosampler, vapour generator, atomic fluorescence spectrometer and control computer.*

(see equation 3). Mercury is atomic and a vapour at room temperature. The interface between the vapour generator and the detector provides a flow of mercury vapour in an argon stream which can also be sheathed in a further flow of argon.

This provides an efficient transfer of samples into the Merlin and most importantly a rapid flush-out. The interface does not provide any of the hold up of mercury, which is a feature of the commonly used atomic absorption technique. To aid the transfer of mercury vapour the tin (II) chloride regime is used with a specific gas/liquid separator designed for this task. Mercury is effectively sparged from the reaction vessel into the Merlin detector. Full automation is provided using a simple standard Digital Input Output (DIO) card fitted into an IBM compatible computer system with suitable software. This is an easy-to-use menu driven system which controls the modules used in the Merlin Plus, calibrates the system, collects, collates and reprints the results and which links to further host computer systems.

Reagents, standards and solutions are prepared according to the procedures laid out by the *Yorkshire Water Methods of Analysis*. Care must be taken to minimize the blank levels of mercury present.

*Sample collection and preservation*

Samples are taken in 300 ml disposable paper cups after pre-rinsing with the sample twice. An aliquot of the sample is then immediately transferred from the paper cup to a clean 100 ml measuring cylinder containing 15 ml of 33% V/V hydrochloride acid and 2 ml of 0·167 ml $KBrO_3/KBr$. The cylinder is filled to a volume between 90 and 100 ml. The total occupied volume is then recorded on the cylinder label, together with the sampling point details. If a filtered mercury sample is required, the filtration should be carried out as soon as possible after sampling. The filtrate is treated as above.

The containers then stand for at least one hour to ensure complete breakdown of organomercury compounds. If a distinct bromine colour remains, the sample can be stored for at least two days if mercury contamination is avoided.

If the sample contains much organic matter and no free bromine remains, a further 2 ml addition of the potassium bromate/bromide reagent is made. In this instance, a blank containing twice the normal amount of this reagent is also prepared.

*Operation*

Reagents and standards are prepared as above. The Merlin Plus is set up according to the manufacturer's

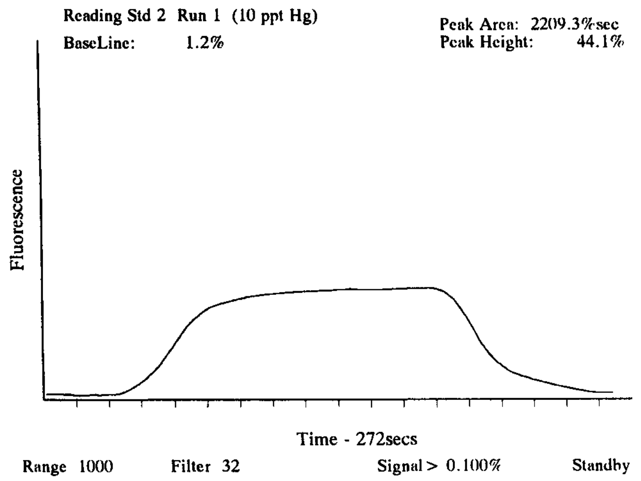

*Figure 4. Fluorescence signal for a 10 ppt standard, showing characteristic peak shape and sensitivity of system.*

recommendations and allowed to stabilize for 30 min. A quick check with coloured solutions will ensure that the equipment is functioning correctly. The reagents blank and samples are then presented to the vapour generator. Argon is the preferred transfer gas, since nitrogen and air will quench the fluorescence and reduce sensitivity by 8- and 30-fold respectively. However, this feature can be used to extend the linear range where high concentrations of mercury are present.

The Merlin Plus System is then calibrated over the range of interest using the software to prompt and store the results. A typical signal response is shown in figure 4. This represents the output typically obtained from 0.010 ppb of mercury in water. The shape of the peak also provides valuable diagnostic data on the analysis, and change in this shape will either indicate an interference or a minor malfunction of the equipment. Good peaks are consistent with good data. The calibration graphs are computed by the method of least squares and show excellent correlations and confirm the linearity and sensitivity of the method and Merlin's applicability to mercury analyses. Where blanks are matched closely to the samples, the graphs are directed through the origin as expected and the equal weight slope average method of calculation is preferred. P S Analytical's Merlin Plus provides a rapid sensitive fully automatic system for mercury analysis at ultra trace levels. It can be applied to a wide range of samples and systems have been installed in both laboratory and process control applications.

*Commentary*

The method uses a continuous flow approach for the analysis of the trace mercury levels. This was chosen as it provides the best analytical data and because it will analyse samples at a considerably high rate. Since the chemistry is reaching a steady-state procedure—the kinetics of the reaction are indicative of any interference that are present. A batch or flow injection approach will not provide this additional information. Table 1 shows some of the advantages and disadvantages of the method of automation. The chemistry of the reaction, i.e. conversion of mercury compounds into mercury vapour, is optimized using a tin (II) chloride reagent system. Normally acidic solutions are preferred, however. There are some advantages in the use of alkaline conditions for particular matrix applications.

Current legislation requires the analysis of total mercury. In one sense this presents a problem since only inorganic mercury will react with the tin (II) chloride. However, in conjunction with Yorkshire Water LabServices, PSA has developed a suitable technique to convert all organo mercury species into the inorganic form using a bromate–bromide reagent. This reagent solution in itself represents a facet of automation strategy. Equation 2 clearly shows that by reducing the chemist's activity in an operation reduces the costs. In this situation the reagent is added to the sample at the time it is collected, hence when the sample is received in the laboratory it is both converted into the correct form for analysis and it is also preserved for several days. Thus two problems are overcome: the use of complicated instrumentation to transfer the organo mercury into the inorganic form is avoided; and the level of mercury is fixed for analysis.

In routine operation it is possible to generate a level of moisture in the analytical stream. The use of the Perma Pure drier tube (Perma Pure Products, Monmouth Airport, Farmingdale, NJ 07727, USA) continuously removes the moisture in an online fashion, maintaining the instrument performance. Moisture traps, on the other hand, will often clog up and become ineffective. The optimization of the chemistry, the sample pretreatment and the detector itself allows the user to provide analytical data which can be categorically stated to be mercury at a defined level in the parts per trillion level. Figure 4 shows a typical signal for 10 ppt, which clearly illustrates the overall performance levels of the method and instrument used.

*Table 1.*

| | Continuous flow | Batch analysis |
|---|---|---|
| Advantages | • Precise control over reaction conditions<br>• Constant generation of hydrogen<br>• Experienced operators not required<br>• Precisions of approximately 1% easily obtainable in linear range | • Small sample requirement<br>• Economical reagent usage<br>• Inexpensive equipment |
| Disadvantages | • Large sample volume required<br>• Long analysis time 60 sec | • Operator intensive<br>• Precision is function of injection technique<br>• Intermittent production of hydrogen<br>• Time consuming |

### Conclusions

The role of the analyst in laboratory automation is particularly exciting and important. To enable a full contribution, the analyst must be conversant with a wide range of skills, from analytical chemistry through to computer applications. Above all, they must have a flexible approach and be able to communicate both to their peers and their customers.

